# Bone Allograft Acid Lysates Change the Genetic Signature of Gingival Fibroblasts

**DOI:** 10.3390/ijms242216181

**Published:** 2023-11-10

**Authors:** Layla Panahipour, Azarakhsh Oladzad Abbasabadi, Anja Wagner, Klaus Kratochwill, Monika Pichler, Reinhard Gruber

**Affiliations:** 1 Department of Oral Biology, University Clinic of Dentistry, Medical University of Vienna, 1090 Vienna, Austria; layla.panahipour@meduniwien.ac.at (L.P.); az.azar66@gmail.com (A.O.A.); 2 Core Facility Proteomics, Medical University of Vienna, 1090 Vienna, Austria; anja.wagner@meduniwien.ac.at (A.W.); klaus.kratochwill@meduniwien.ac.at (K.K.); 3 Christian Doppler Laboratory for Molecular Stress Research in Peritoneal Dialysis, Department of Pediatrics and Adolescent Medicine, Medical University of Vienna, 1090 Vienna, Austria; 4 Division of Pediatric Nephrology and Gastroenterology, Department of Pediatrics and Adolescent Medicine, Comprehensive Center for Pediatrics, Medical University of Vienna, 1090 Vienna, Austria; 5 Cells + Tissuebank Austria, 3500 Krems, Austria; m.pichler@ctba.at; 6 Department of Periodontology, School of Dental Medicine, University of Bern, 3010 Bern, Switzerland; 7 Austrian Cluster for Tissue Regeneration, 1200 Vienna, Austria

**Keywords:** bone allograft, RNAseq, gingival fibroblasts, bone regeneration, bone augmentation, IL11, AREG, C11orf96

## Abstract

Bone allografts are widely used as osteoconductive support to guide bone regrowth. Bone allografts are more than a scaffold for the immigrating cells as they maintain some bioactivity of the original bone matrix. Yet, it remains unclear how immigrating cells respond to bone allografts. To this end, we have evaluated the response of mesenchymal cells exposed to acid lysates of bone allografts (ALBA). RNAseq revealed that ALBA has a strong impact on the genetic signature of gingival fibroblasts, indicated by the increased expression of IL11, AREG, C11orf96, STC1, and GK—as confirmed by RT-PCR, and for IL11 and STC1 by immunoassays. Considering that transforming growth factor-β (TGF-β) is stored in the bone matrix and may have caused the expression changes, we performed a proteomics analysis, TGF-β immunoassay, and smad2/3 nuclear translocation. ALBA neither showed detectable TGF-β nor was the lysate able to induce smad2/3 translocation. Nevertheless, the TGF-β receptor type I kinase inhibitor SB431542 significantly decreased the expression of IL11, AREG, and C11orf96, suggesting that other agonists than TGF-β are responsible for the robust cell response. The findings suggest that IL11, AREG, and C11orf96 expression in mesenchymal cells can serve as a bioassay reflecting the bioactivity of the bone allografts.

## 1. Introduction

Bone is a living tissue with an amazing ability to heal [1] but larger defects that exceed a critical size [2], smaller defects undergoing inflammatory osteolysis [3], and bone atrophy [4], particularly in elderly patients [5], sometimes refuse to heal. Considering the growing elderly population with their need for reconstructive surgery in orthopedic [5] or dental procedures and maxillofacial reconstructions [6], there is a concomitant demand for bone grafts that require osteoconductive properties that allow new bone to be laid down on the graft surface and, hence, guide new bone into the reconstructed site [7]. However, the harvesting of autologous bone, particularly for mandibular reconstructions [8] but also mandibular symphysis, retromolar regions, and ramus [9,10], is an invasive procedure associated with morbidity and the volume to be harvested is limited. Moreover, the original volume achieved upon augmentation with autologous bone shrinks [11], thus resorption of the grafts requires over-augmentation and combination with slow-resorbing biomaterials [12]. Thus, and because only a limited amount of autologous bone grafts can be harvested from the same patient to be treated, allogenic bone grafts prepared from the femoral head of a patient undergoing hip replacement, multi-organ donors, or even post-mortem donors are more accessible [13]. Allogenic bone grafts, however, require processing, a multistep process that might disturb the biological activity of the original donor bone [13]. Thus, it is reasonable to raise questions about the biological activity remaining in allogenic bone after processing. 

If there is a biological activity remaining in allogenic bone, it might be caused by acid- and temperature-stable growth factors such as TGF-β1 [14] and bone morphogenetic proteins (BMPs) [15]. These growth factors are presumably released during the catabolic phase of graft consolidation and somehow affect the overall process of bone regeneration [16]. Even though this assumption is reasonable, evidence to support this claim is mainly based on bioassays. For instance, TGF-β identified in acid lysates of native bovine bone caused a robust increase in IL11 expression and other classical TGF-β1 target genes [17,18]. However, the clinical implication of high IL11 levels is indirect even though IL11 plays a critical role in regeneration and may exert fibrotic activity [19,20]. Apart from IL11, amphiregulin (AREG), being relevant for immunity, inflammation, and tissue repair [21], was identified by our previous RNAseq approach where gingival fibroblasts were exposed to acid lysates of vital bone [17]. The robust cell response to acid lysates of vital bone has prompted us to study the cell response to lysates prepared from allografts. 

Allogenic demineralized bone can be a vital source of TGF-β1 and, consequently, aqueous solutions and acid lysates thereof increase the expression of IL11 and other target genes in gingival fibroblasts [22]. Also, our laboratory-based demineralized bone matrix as well as extracts of commercial allografts were capable of raising IL11 expression in gingival fibroblasts [23]. Defatting with acetone and methanol lowered the capacity of the demineralized bone matrix to drive IL11 expression, suggesting that the processing of bone affects its biologic activity [23]. Manufacturers of bone allografts have refined their protocols for processing fresh bone with the aim of inactivate and remove risky agents, and reduce the risk of disease transmission. Detergents, ethanol, acetone, and ether as well as hydrogen peroxide are effective for bleaching and eliminating viral and bacterial loads [13,24]. One important question is related to the activity remaining in a bone allograft.

The aim of the present research is to refine our biological understanding of bone allografts based on analyzing the cell response to acid lysates of bone allografts (ALBA) by implementing RNAseq to identify sensitive target genes and to understand the possible involvement of TGF-β1 in the regulation of gene expression. 

## 2. Results

### 2.1. ALBA Changes the Genetic Signature of Gingival Fibroblasts

**Figure 1:**
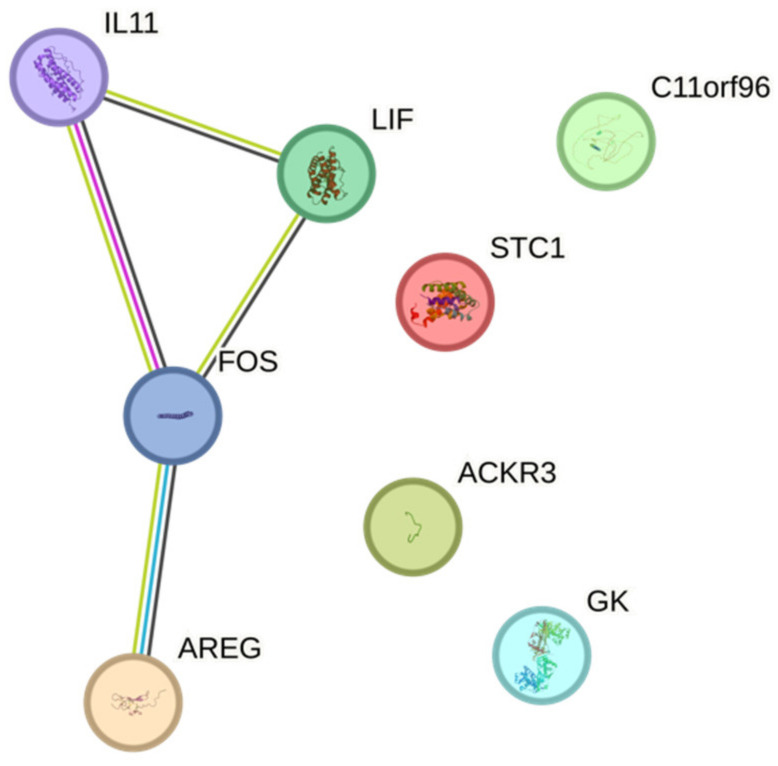
RNAseq analysis of gingival fibroblasts exposed to ALBA. Gingival fibroblasts were exposed to acid lysates of bone allografts (ALBA). RNAseq revealed a panel of highly regulated genes (>50-fold) that are considered biomarkers to reflect the response of local mesenchymal cells. Data are shown by STRING analysis.

**Figure 2:**
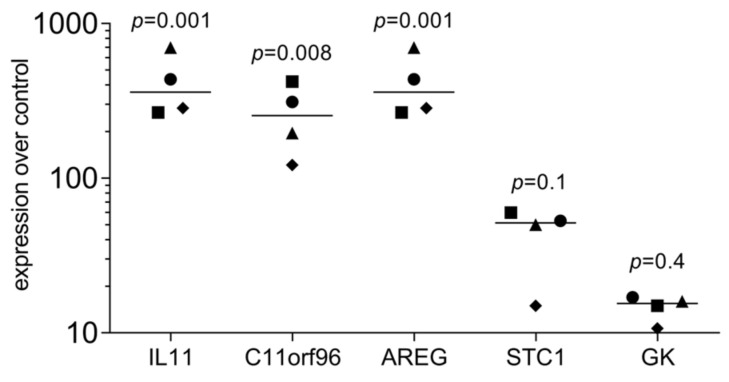
RT-PCR analysis of gingival fibroblasts exposed to ALBA. Gingival fibroblasts were incubated with acid lysates of bone allografts (ALBA). RT-PCR analysis confirmed the strong increase in expression changes in ALBA-induced gingival fibroblasts. Data points represent four independent experiments. Data were normalized to untreated control cells with x-fold changes compared to the untreated cells. The statistical analysis was based on the Friedmann test with uncorrected Dunn’s test.

To understand the impact of ALBA on gingival fibroblasts, an RNAseq approach was performed. RNAseq revealed a robust change in the signature with IL11, AREG, C11orf96, STC1, GK, LIF, FOS, and ACKR3 being more than 50-fold upregulated (Figure 1, Supplement Table S1). We also have identified a sharp increase in EREG, IL24, CREB5, PDE10A, CCR7, CMYA5, NOX4, NTN1, ACKR4, and SHISA2 with no detectable gene counts in the untreated cells (Supplement Table S1). OSR1, ADH1B, TBKBP1, and OMD were at least 20-fold downregulated (Supplement Table S1). If we consider all the genes with at least a 20-fold change in a STRING analysis, we see enrichment for cytokine–cytokine receptor interaction (hsa04060; 6 out of 282 genes) and, for instance, positive regulation of cell population proliferation (GO 0008284; 11 out of 945 genes). The GO analysis and the KEGG pathway analysis are indicated in Supplement Table S1. Next, we performed a single gene expression analysis of gingival fibroblasts exposed to ALBA. We could establish a protocol verifying the robust increase in IL11, AREG, and C11orf96, while STC1 and GK expression showed a trend but failed to reach the level of significance (Figure 2). 

### 2.2. TGF-β RI Kinase Inhibitor Reduced ALBA-Induced Expression of IL11, AREG, C11orf96

**Figure 3:**
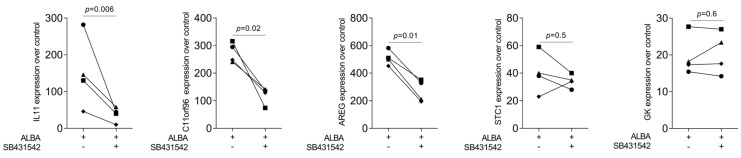
RT-PCR analysis of gingival fibroblasts in the presence of SB431542. RT-PCR analysis of gingival fibroblasts incubated with acid lysates of bone allograft (ALBA) with and without the TGF-β RI kinase inhibitor SB431542. Expression analysis showed that the blocking of TGF-β signaling reduced ALBA-induced expression of IL11, AREG, and C11orf96 in gingival fibroblasts. Data points represent four independent experiments. Data were normalized against untreated control cells with x-fold changes compared to the untreated cells. The analysis was based on a paired *t*-test.

**Figure 4:**
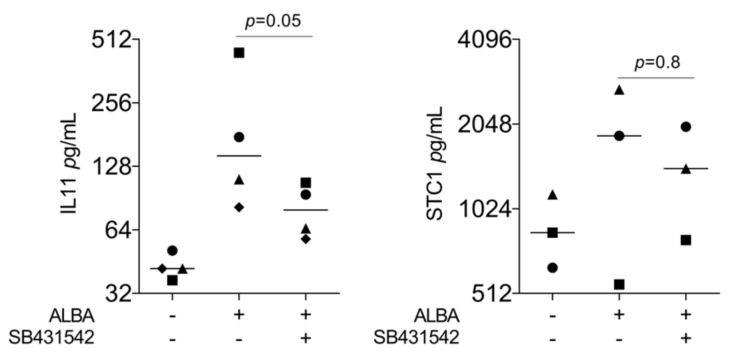
Immunoassay analysis of gingival fibroblasts in the presence of SB431542. Immunoassay of gingival fibroblasts exposed to acid lysates of bone allograft (ALBA) with and without the TGF-β RI kinase inhibitor SB431542. Immunoassay indicated that blocking of TGF-β signaling reduced ALBA-induced expression of IL11 but not STC1 in gingival fibroblasts. Data points represent four and three independent experiments, respectively. The analysis was based on a ratio-paired *t*-test.

**Figure 5:**
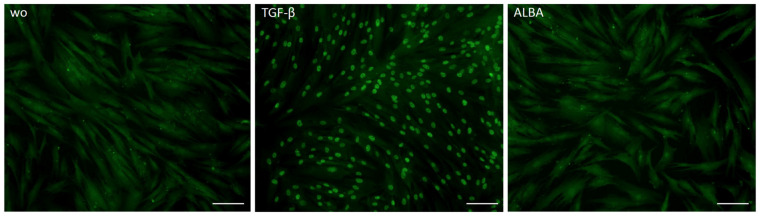
Immunostaining of gingival fibroblasts for smad2/3 nuclear translocation. Immunostaining of gingival fibroblasts exposed to acid lysates of bone allograft (ALBA) or TGF-β1. The staining pattern revealed a strong nuclear translocation of smad2/3 when gingival fibroblasts are exposed to recombinant TGF-β1 but not to ALBA. The scale bars represent 100 µm.

Considering that IL11 [22] and AREG [25] are classical TGF-β target genes and lysates of the demineralized bone matrix contain TGF-β [22], we have tested the hypothesis that the increase in our gene panel is mediated by TGF-β signaling. Indeed, the TGF-β RI kinase inhibitor SB431542 significantly reduced ALBA-induced expression of IL11, AREG, and C11orf96—but not of STC1 and GK (Figure 3). This observation was further confirmed at the protein level where IL11 but not STC1 was reduced by SB431542 (Figure 4). However, in contrast to recombinant TGF-β1, ALBA failed to provoke the nuclear translocation of smad2/3 in gingival fibroblasts (Figure 5). 

### 2.3. Proteomic Analysis and Immunoassay Failed to Identify TGF-β

**Figure 6:**
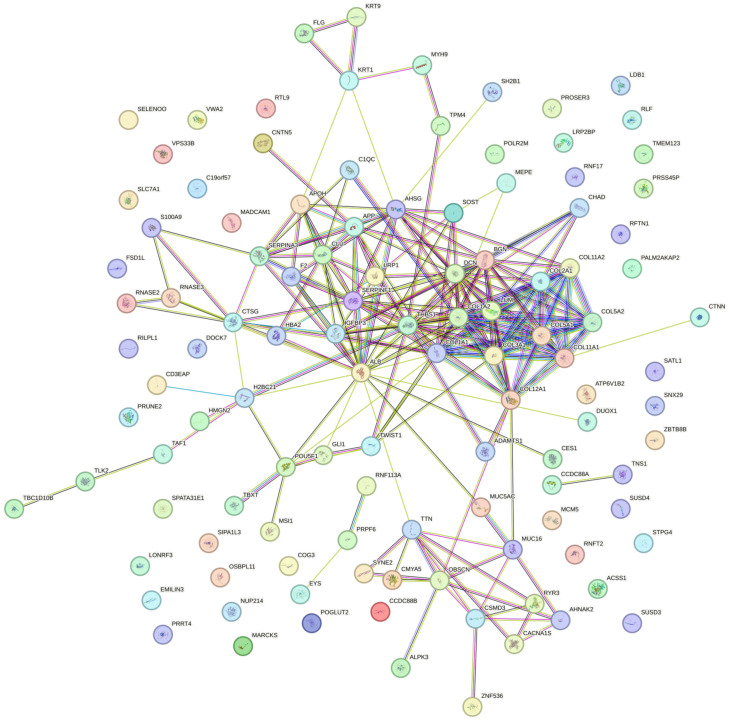
STRING analysis of bone allograft proteome. STRING analysis of the 114 proteins common to all batches identified. Please note the cluster related to the collagen matrix and non-collagenous proteins.

To identify TGF-β in ALBA, we have performed an immunoassay but failed to detect the growth factor. We then performed proteomics of the original bone allograft. We identified 114 proteins that were detectable in all three batches of bone allografts (Figure 6; Supplement Table S2). Among the proteins that form a cluster are COL1A1, COL1A2, COL2A1, COL3A1, COL5A1, COL5A2, COL11A1, COL11A2, COL12A1, COL22A1, and LUM involved in collagen chain trimerization (CL:19468; 11 out of 32), and banded collagen fibril (CL:19471; 9 out of 10). Apart from their structural function, COL1A1, COL1A2, COL2A1, COL3A1, and COL5A1 but also THBS1 and IGFBP3 are linked to growth factor binding (GO:0019838, 7 out of 127). Concerning growth factors, we identified BMP6, FGF2, FGF20, and HDGF but not TGF-β in independent batches (Supplement Table S2). A summary of the enriched terms is presented in Supplement Table S2. 

## 3. Discussion

Allograft research is usually performed by clinical examination where the bone grafts are placed in a defect site and the endpoints are related to graft consolidation, the success rate of dental implants, or the amount of bone that has formed [26,27]. Clinical research with bone allografts is relevant but the underlying cellular and molecular responses remain hidden. A complementary approach to clinical research is studying the in vitro cell response to fractions of bone allografts; this bioassay at least partially simulates the complex clinical situation of a defect site [22,23]. Even though clinical research and bioassays have their strengths and limitations, we should appreciate their value in better understanding the biology of bone allografts, in particular because bone allografts undergo processing and are consequently not standardized. Moreover, the various protocols of allograft processing are intellectual properties of companies, hence research findings obtained with one bone allograft are not necessarily valid for those from another provider. Sensitive bioassays are thus required to show the biological properties of bone allografts from different batches or companies. 

The bioassay approach presented here considers the experience we have gained from analyzing acid bone [17,18,28] and dentine lysates [29,30,31] as well as bone-conditioned medium [32], overall showing that IL11 is a sensitive gene that is strongly regulated in gingival fibroblasts exposed to lysates and conditioned medium. IL11 is presumably more than just a biomarker as IL11 plays a central role in mediating the fibrotic activity of TGF-β [19,20], for instance, in cardiovascular and pulmonary fibrosis [33,34] as well as systemic sclerosis [35], non-alcoholic steatohepatitis [36], and kidney dysfunction [37]. Conversely, IL11 is a mediator of bone modeling [38], IL11 content was 450-fold higher in human fracture hematoma when compared to peripheral blood [39] and rodent models support the early 70-fold increase of IL11 in fracture healing [40], supporting its physiological function in bone homeostasis and presumably graft consolidation. Considering that at sites of early bone regeneration a provisional extracellular matrix is produced by an as yet not well-characterized fibroblastic population [41], a role of endogenously produced IL11 to support bone regeneration can be hypothesized. Thus, at the moment, we can discuss our findings at the level of a hypothesis but the sharp increase in IL11 expression driven by ALBA is a solid primer for future research.

In accordance with our previous research [17,18,28,29,30,31], blocking TGF-β receptor type I kinase with the inhibitor SB431542 reduced the expression of IL11, similar to what we have observed with AREG and C11orf96. However, we cannot blame allograft-derived TGF-β for mediating the increase in IL11, AREG, and C11orf96—first, we could not identify TGF-β by immunoassay and proteomics, and second, ALBA failed to provoke the activation of the canonical TGF-β signaling pathway culminating in the nuclear translocation of smad2/3. Thus, other ligands than TGF-β in bone allografts, for instance, activins [42,43], may have the potential to drive the expression of IL11, AREG, and C11orf96 in mesenchymal cells, knowing that SB431542 is an inhibitor of activin and TGF-β signaling [44]. However, we could not identify activin-A by proteomic analysis. Also, proteins related to growth factor binding might play roles in mediating the effects of ALBA but this aspect is a task for future research. It is thus possible that other pathways than canonical TGF-β signaling mediate the ALBA-induced expression of IL11, AREG, and C11orf96. For instance, IL11 expression is sensitive to oxidative stress [45], mechanical damage of chondrocyte micromass cultures [46], and traumatic brain injury [47], suggesting that damage-associated molecular patterns (DAMPs), as they are presumably present in bone allografts [48], might drive IL11 expression. Moreover, several functional elements implicated in the induction of AREG expression have been identified in the promoter [49] and AREG expression is induced through the activation of various pathways, including cAMP/PKA and protein kinase C [49]. Future research should aim to explore the possibility that DAMPs within ALBA mediate the change in the signature in gingival fibroblasts and other cells of the mesenchymal lineage. 

Apart from IL11, also AREG, C11orf96, STC1, and GK were also among the strongly regulated genes induced by ALBA, all supposedly having a role in bone biology. For example, AREG supports bone formation, as transgenic mice overexpressing AREG in osteoblasts had a transiently increased trabecular bone mass [50] and AREG knockout mice have a mild decrease in bone mineral density and cortical thickness at the femoral midshaft [51]. Thus, while accumulating evidence supports the role of IL11 and AREG in bone biology, the role of C11orf96 needs to be discovered [52]. More information is available for STC1; its overexposure inhibits normal skeletal development in mice [53], a fibroblast-derived paracrine STC1 targets macrophages [54], and STC1 protects endothelial cells from inflammatory injury [55]. Moreover, mutations and phenotypes with isolated GK deficiency suffer from bone dysplasia and growth delay [56,57]. Taken together, at least theoretically, ALBA can increase genes which may change the local environment of the recipient site of bone allografts. 

The study is pilot in nature and has limitations. Our ALBA is an acid lysate of the original bone allograft; thus, a pH-dependent soluble fraction was used to study gene expression changes. It remains unclear if the remaining demineralized bone allograft matrix has similar bioactivity and may even modulate the genetic signature of cells differently than ALBA. Moreover, the choice of 1.0 M HCl is critical, as this strong acid concentration might not simulate the biological environment accurately. Indeed, the pH the osteoclasts use to demineralize the bone is around pH = 4.5 [58], hence it was by far too high to simulate the physiological scenario. Considering this limitation, 1.0 M HCl was used to demineralize the dentine matrix based on previous bioassays [29] and, in vitro, TGF-β is activated by low pH, lower than the osteoclasts provide [59]. Nevertheless, we are indeed at risk of losing the activity of other potential bioactive molecules that are in ALBA—however, in the proteomics analysis, which is independent of the low pH, we could not consistently identify growth factors and we were not able to detect TGF-β. Moreover, HCl treatment durations differed between cell culture and proteomic research. We have decided to use the total lysis for allograft proteomics to not overlook potential growth factors not released by 1.0 M HCl. In future studies, however, we may include ALBA in the proteomic analyses.

Another limitation is that we have restricted our analysis to gingival fibroblasts that have an osteogenic potential but do not fully represent a bony environment [60]. Moreover, gingival fibroblast and osteogenic cells, usually show a comparable cell response. Interestingly, however, in our preliminary experiments, neither the osteosarcoma-derived cell line MG63 nor the fetal osteoblastic cell line hFOB 1.19 [61] showed an increased IL11 expression when exposed to ALBA or recombinant TGF-β. Future research should therefore confirm the ALBA-induced expression of IL11, AREG, and C11orf96 with cells responsive to TGF-β such as primary bone cells [62]. It is likely that primary bone-derived fibroblasts respond to ALBA with an increased IL11 expression as, in principle, osteogenic cells show a robust response to TGF-β and IL11 is a TGF-β target gene, independent of the cell type. The main reason for having used gingival fibroblasts here is that these cells are well established in our laboratory, ethically approved, and turned out to be a robust bioassay to determine TGF-β activity—measured by IL11 expression [17]. Moreover, in the future, we have to analyze the impact of ALBA on EREG, IL24, and the other genes being strongly increased in the RNAseq but not detected in untreated cells. For instance, IL24 is a multifunctional cytokine with complex regulatory functions [63] and EREG acts as an EGF receptor agonist, similar to AREG [64]. Future research should further consider the impact of ALBA on other cell types required for bone regeneration such as bone-resorbing osteoclasts, macrophages, vascular endothelial cells, and even lymphocytes, ideally including an RNAseq screening approach. 

The central question that arises is related to the molecular signal originating from bone allografts being responsible for the dramatic increase in IL11, AREG, and C11orf96 expression. The signaling pathways driving the expression changes should be discovered to see if they are similar or different, which is likely as IL11, AREG, and C11orf96—and STC1 and GK—show a different expression characteristic and an SB431542 dependency. Additionally, from a technical aspect, understanding the molecular mechanism of how ALBA drives gene expression in target cells would be a basis for monitoring the manufacturing process of bone allografts, for instance, the impact of defatting [65] and virus inactivation [66]. Overall, our bioassay might help to validate the activity of bone allografts. However, we have to be aware that the strong changes in IL11, AREG, and C11orf96 expression are not necessarily surrogate parameters predicting the clinical success or the consolidation of the bone allografts. Thus, the clinical relevance of our observations remains an open research question.

Taken together, we report here our findings from a screening approach aiming to understand the biological activity of bone allografts where we succeeded in identifying IL11, AREG, and C11orf96 as sensitive target genes induced by ALBA in fibroblasts. 

## 4. Material and Methods

### 4.1. Preparation of Acid Lysates of Bone Allografts (ALBA)

For the bone allograft (Maxgraft^®^; cortico-cancellous allograft bone substitute from human donor bone; Cells+ Tissuebank Austria GmbH, Krems, Austria), three batches of mineralized bone granules were used, X21082; X22003; F21018. Bone allografts at 400 mg/mL with 1.0 M HCl were stirred overnight in a glass beaker at room temperature. Acid lysates were harvested and centrifuged at 20,000 RCF for five minutes. The pH of the lysate was neutralized with 10.0 M NaOH and sterile filtered (0.2 µm, VWR International, Radnor, PA, USA). For desalting, the lysates were subjected to a PD SpinTrap G-25 microspin column designed for desalting and buffer exchange of biological samples (Sigma Aldrich, St. Louis, MO, USA). If not used immediately, lysates were stored in aliquots at −20 °C. The acid lysates of the bone allograft (ALBA) were prepared individually from each lot and used at a concentration of 30% in cell culture experiments. 

### 4.2. Proteomics Sample Preparation

A total of 100 mg of bone allograft samples (3 different lots) was incubated with 1 mL 1 M HCl for 72 h at room temperature on a thermomixer shaking at 800 rpm. Afterward, samples were neutralized with 10 M NaOH, centrifuged for 3 min at 10,000× *g*, and the supernatant was collected. Then, 650 µL of lysis buffer (30 mM Tris, pH 8.5, 7 M urea, 2 M thiourea, 4% CHAPS, 1 mM EDTA, one tablet of Complete Protease Inhibitor (Roche, Basel, Switzerland) per 100 mL, and one tablet of PhosStop Protease Inhibitor (Roche) per 100 mL) was added to each sample and they were stored at −20 °C until further use. Samples were concentrated and the buffer was exchanged using Amicon filter units with a 3 K cut-off (Millipore, Burlington, MA, USA) according to the manual. Briefly, the samples were loaded onto the filter, centrifuged, and washed 2 times with 300 µL lysis buffer until a final volume of 150–180 µL was reached. Protein content was measured using a Pierce 660 nm assay (Thermo Fisher Scientific, Waltham, MA, USA). First, 30 µg of each sample was digested using single-pot, solid-phase enhanced sample preparation (SP3). Briefly, the reduced (10 mM DTT for 1 h at 56 °C) and alkylated (55 mM IAA, 30 min at RT) proteins were bound to SP3 beads (10:1 beads: protein ratio, GE Healthcare, Chicago, IL, USA), washed with 80% ethanol and acetonitrile, and subjected to on-bead digestion with trypsin/LysC Mix (1:25 protease: protein ratio, Promega) overnight at 37 °C in 50 mM ammonium bicarbonate, pH 8.5. After elution, peptides were desalted using Pierce Peptide Desalting spin columns (Thermo Fisher Scientific, Waltham, MA, USA) according to the manual. The elutions were dried in a vacuum concentrator and reconstituted in 0.1% trifluoroacetic acid. All chemicals were purchased from Sigma if not stated otherwise.

### 4.3. LC–MS/MS Analysis

Samples were analyzed on an Ultimate 3000 RSLC nano system directly coupled to an Exploris 480 with FAIMSpro (Thermo Fisher Scientific). Four microliters of each sample was injected onto a reversed-phase C18 column (50 cm × 75 µm i.d., packed in-house with 3 µm particles) with solvent A being 0.4% formic acid in water and solvent B 90% acetonitrile with 0.4% formic acid. Total LC–MS/MS run time per sample was 120 min with a gradient of 4.0% to 38% mobile phase B over 94 min at a flow rate of 230 nL/min at 40 °C. The Exploris 480 was operated in positive ion mode with a spray voltage of 1650 V. MS scans were performed in the range from *m*/*z* 375–1650 at a resolution of 60,000 (at *m*/*z* = 200). MS/MS scans were performed, choosing a resolution of 15,000; normalized collision energy of 29%; isolation width of 1.4 *m/z*; and dynamic exclusion of 90 s. Two different FAIMS voltages were applied (−40 V and −60 V) with a cycle time of 1.5 s per voltage. FAIMS was operated in standard resolution mode with a static carrier gas flow of 4.1 L/min.

### 4.4. Data Processing

The acquired raw MS data files were processed and analyzed using ProteomeDiscoverer (v2.4.0.305, Thermo Fisher). SequestHT was used as a search engine and the following parameters were chosen: database: Homo sapiens (SwissProt, downloaded on 2 February 2023); enzyme: trypsin; max. missed cleavage sites: 2; static modifications: carbamidomethyl (C); dynamic modifications: oxidation (M), acetyl (protein N-terminus), Met-loss (M) and Met-loss + acetyl (M); precursor mass tolerance: 10 ppm; fragment mass tolerance: 0.02 Da. Single peptide IDs of proteins identified with high confidence were retained in the individual LC–MS analyses but only interpreted if found in all three independent replicates, making it highly unlikely that these proteins are false positives identified by chance.

### 4.5. Cell Culture

Gingival fibroblasts were prepared from explants of human gingiva harvested from extracted wisdom teeth of patients who had given informed consent. The harvesting procedure was approved by the Ethics Committee of the Medical University of Vienna (EK NR 631/2007). Gingival fibroblasts were seeded at 30,000 cells/cm^2^ and incubated with 30% ALBA in a serum-free medium for 24 h followed by gene expression analysis. If indicated, 10 μM of the TGF-β RI kinase inhibitor SB431542 (Calbiochem, Merck, Billerica, MA, USA) or 10 ng/mL TGF-β1 (ProSpec-Tany TechnoGene Ltd., Ness-Ziona, Israel) was applied. 

### 4.6. RNAseq

RNA quality was evaluated using the Agilent 2100 Bioanalyzer (Agilent Technologies, Santa Clara, CA, USA). Sequencing libraries were prepared at Genomics Core Facility, Medical University of Vienna using the NEBNext Poly (A) mRNA Magnetic Isolation Module and the NEBNext Ultra™ II Directional RNA Library Prep Kit for Illumina according to the manufacturer’s protocols (New England Biolabs, Ipswich, MA, USA). Libraries were QC-checked on a Bioanalyzer 2100 (Agilent, Santa Clara, CA, USA) using a High Sensitivity DNA Kit for correct insert size and quantified using a Qubit dsDNA HS Assay (Invitrogen, Waltham, MA, USA). Pooled libraries were sequenced on a NextSeq500 instrument (Illumina, San Diego, CA, USA) in 1 × 75 bp single-end sequencing mode. Approximately 25 million reads were generated per sample. Reads in fastq format were aligned to the human reference genome version GRCh38 (www.ncbi.nlm.nih.gov/grc/human, accessed on 10 October 2023) with Gencode 29 annotations (www.gencodegenes.org/human/release_29.html, accessed on 10 October 2023) using STAR aligner 55 version 2.6.1a in 2-pass mode. Reads per gene were counted by STAR, and differential gene expression was calculated. 

### 4.7. Real-Time Polymerase Chain Reaction (RT-PCR) and Immunoassay

Total RNA was isolated with the ExtractMe total RNA kit (Blirt S.A., Gdańsk, Poland), followed by reverse transcription and polymerase chain reaction (LabQ, Labconsulting, Vienna, Austria) on a CFX Connect™ Real-Time PCR Detection System (Bio-Rad Laboratories, Hercules, CA, USA). The mRNA levels were calculated by normalizing to the housekeeping gene GAPDH using the ΔΔCt method. Primer sequences are IL11 F-AAATAAGGCACAGATGCC, R-CCTTCCAAAGCCAGATC; C11orf96 F-TCACGCCAACACTCTCGCTGAA, R-CAATCCTCCAGACGCAGTAGCA; AREG F-GTGGTGCTGTCGCTCTTGATA, R-CCCCAGAAAATGGTTCACGCT; STC1 F-GCAGGAAGAGTGCTACAGCAAG, R-CATTCCAGCAGGCTTCGGACAA; GK F-AGCTCAGCATCCTTGGAAGTGG, R-GAACTGGTCCATGATGCCACAG; GAPDH F-AAGCCACATCGCTCAGACAC, R-GCCCAATACGACCAAATCC. We have normalized the data to the untreated control cells that are “1-fold” by calculation. The other data are x-fold changes compared to the untreated cells. For the immunoassays, the human IL11 and STC1 kit (R&D Systems, Minneapolis, MN, USA) was used. The immunoassay kits were used according to the manufacturer’s instructions. 

### 4.8. Immunofluorescence Analysis

Gingival fibroblasts were plated on Millicell^®^ EZ slides (Merck KGaA, Darmstadt, Germany) and stimulated with 30% ALBA or 10 ng/mL TGF-β1 (ProSpec-Tany TechnoGene Ltd.). Cells were fixed with 4% paraformaldehyde, blocked with 1% bovine serum albumin, and permeabilized with 0.3% Triton X-100 (all Sigma-Aldrich). Smad2/3 (D7G7 XP^®^, Cell Signaling Technology, Danvers, MA, USA) was used overnight at 4 °C. Detection was performed with an Alexa 488 secondary antibody (CS-4412, Cell Signaling Technology). We captured the images on a fluorescence microscope with the DAPI-FITC dual excitation filter block (Echo Revolve Fluorescence Microscope, San Diego, CA, USA).

### 4.9. Statistical Analysis

All experiments were performed at least four times. Statistical analyses were performed with ratio paired *t*-tests or Friedmann test whenever appropriate. Analyses were performed using Prism v.9 (GraphPad Software; San Diego, CA, USA). Significance was set at *p* < 0.05.

## Data Availability

All data are available on demand.

## Supplementary Materials

The following supporting information can be downloaded at: https://www.mdpi.com/article/10.3390/ijms242216181/s1.

## References

[1] Einhorn T.A., Gerstenfeld L.C. (2015). Fracture healing: Mechanisms and interventions. Nat. Rev. Rheumatol..

[2] Schmitz J.P., Hollinger J.O. (1986). The critical size defect as an experimental model for craniomandibulofacial nonunions. Clin. Orthop. Relat. Res..

[3] Apaza Alccayhuaman K.A., Heimel P., Lee J.S., Tangl S., Kuchler U., Marchesan J., Panahipour L., Lettner S., Matalova E., Gruber R. (2023). FasL is a catabolic factor in alveolar bone homeostasis. J. Clin. Periodontol..

[4] Elnayef B., Monje A., Gargallo-Albiol J., Galindo-Moreno P., Wang H.L., Hernandez-Alfaro F. (2017). Vertical Ridge Augmentation in the Atrophic Mandible: A Systematic Review and Meta-Analysis. Int. J. Oral. Maxillofac. Implants.

[5] Gruber R., Koch H., Doll B.A., Tegtmeier F., Einhorn T.A., Hollinger J.O. (2006). Fracture healing in the elderly patient. Exp. Gerontol..

[6] Patel K., Salman S., Shanti R.M. (2023). Bone Allografts: Their Role in Mandibular Reconstruction. Atlas Oral. Maxillofac. Surg. Clin. N. Am..

[7] Busenlechner D., Huber C.D., Vasak C., Dobsak A., Gruber R., Watzek G. (2009). Sinus augmentation analysis revised: The gradient of graft consolidation. Clin. Oral. Implant. Res..

[8] Gu Y., Ma H., Shujaat S., Orhan K., Coucke W., Amoli M.S., Bila M., Politis C., Jacobs R. (2021). Donor- and recipient-site morbidity of vascularized fibular and iliac flaps for mandibular reconstruction: A systematic review and meta-analysis. J. Plast. Reconstr. Aesthet. Surg..

[9] Starch-Jensen T., Deluiz D., Deb S., Bruun N.H., Tinoco E.M.B. (2020). Harvesting of Autogenous Bone Graft from the Ascending Mandibular Ramus Compared with the Chin Region: A Systematic Review and Meta-Analysis Focusing on Complications and Donor Site Morbidity. J. Oral. Maxillofac. Res..

[10] Pereira R.S., Pavelski M.D., Griza G.L., Boos F., Hochuli-Vieira E. (2019). Prospective evaluation of morbidity in patients who underwent autogenous bone-graft harvesting from the mandibular symphysis and retromolar regions. Clin. Implant Dent. Relat. Res..

[11] Elnayef B., Porta C., Suarez-Lopez Del Amo F., Mordini L., Gargallo-Albiol J., Hernandez-Alfaro F. (2018). The Fate of Lateral Ridge Augmentation: A Systematic Review and Meta-Analysis. Int. J. Oral. Maxillofac. Implant..

[12] Miron R.J. (2023). Optimized bone grafting. Periodontol. 2000.

[13] Delloye C., Cornu O., Druez V., Barbier O. (2007). Bone allografts: What they can offer and what they cannot. J. Bone Joint Surg. Br..

[14] Pfeilschifter J., Diel I., Scheppach B., Bretz A., Krempien R., Erdmann J., Schmid G., Reske N., Bismar H., Seck T. (1998). Concentration of transforming growth factor beta in human bone tissue: Relationship to age, menopause, bone turnover, and bone volume. J. Bone Miner. Res..

[15] Celeste A.J., Iannazzi J.A., Taylor R.C., Hewick R.M., Rosen V., Wang E.A., Wozney J.M. (1990). Identification of transforming growth factor beta family members present in bone-inductive protein purified from bovine bone. Proc. Natl. Acad. Sci. USA.

[16] Saulacic N., Bosshardt D.D., Jensen S.S., Miron R.J., Gruber R., Buser D. (2015). Impact of bone graft harvesting techniques on bone formation and graft resorption: A histomorphometric study in the mandibles of minipigs. Clin. Oral. Implant. Res..

[17] Strauss F.J., Stahli A., Beer L., Mitulovic G., Gilmozzi V., Haspel N., Schwab G., Gruber R. (2018). Acid bone lysate activates TGFbeta signalling in human oral fibroblasts. Sci. Rep..

[18] Strauss F.J., Di Summa F., Stahli A., Matos L., Vaca F., Schuldt G., Gruber R. (2019). TGF-beta activity in acid bone lysate adsorbs to titanium surface. Clin. Implant Dent. Relat. Res..

[19] Cook S.A. (2023). The Pathobiology of Interleukin 11 in Mammalian Disease is Likely Explained by its Essential Evolutionary Role for Fin Regeneration. J. Cardiovasc. Transl. Res..

[20] Cook S.A., Schafer S. (2020). Hiding in Plain Sight: Interleukin-11 Emerges as a Master Regulator of Fibrosis, Tissue Integrity, and Stromal Inflammation. Annu. Rev. Med..

[21] Zaiss D.M.W., Gause W.C., Osborne L.C., Artis D. (2015). Emerging functions of amphiregulin in orchestrating immunity, inflammation, and tissue repair. Immunity.

[22] Panahipour L., Omerbasic A., Nasirzade J., Gruber R. (2021). TGF-beta Activity of a Demineralized Bone Matrix. Int. J. Mol. Sci..

[23] Filho G.S., Caballe-Serrano J., Sawada K., Bosshardt D.D., Bianchini M.A., Buser D., Gruber R. (2015). Conditioned medium of demineralized freeze-dried bone activates gene expression in periodontal fibroblasts in vitro. J. Periodontol..

[24] Gruskin E., Doll B.A., Futrell F.W., Schmitz J.P., Hollinger J.O. (2012). Demineralized bone matrix in bone repair: History and use. Adv. Drug Deliv. Rev..

[25] Zhou Y., Lee J.Y., Lee C.M., Cho W.K., Kang M.J., Koff J.L., Yoon P.O., Chae J., Park H.O., Elias J.A. (2012). Amphiregulin, an epidermal growth factor receptor ligand, plays an essential role in the pathogenesis of transforming growth factor-beta-induced pulmonary fibrosis. J. Biol. Chem..

[26] Kloss F.R., Kammerer P.W., Kloss-Brandstatter A. (2022). Risk Factors for Complications Following Staged Alveolar Ridge Augmentation and Dental Implantation: A Retrospective Evaluation of 151 Cases with Allogeneic and 70 Cases with Autogenous Bone Blocks. J. Clin. Med..

[27] Motamedian S.R., Khojaste M., Khojasteh A. (2016). Success rate of implants placed in autogenous bone blocks versus allogenic bone blocks: A systematic literature review. Ann. Maxillofac. Surg..

[28] Strauss F.J., Kuchler U., Kobatake R., Heimel P., Tangl S., Gruber R. (2021). Acid bone lysates reduce bone regeneration in rat calvaria defects. J. Biomed. Mater. Res. A.

[29] Nasirzade J., Kargarpour Z., Panahipour L., Gruber R. (2021). Acid Dentin Lysate Modulates Macrophage Polarization and Osteoclastogenesis In Vitro. Materials.

[30] Nasirzade J., Kargarpour Z., Mitulovic G., Strauss F.J., Panahipour L., Schwarz F., Gruber R. (2021). Proteomic and genomic analysis of acid dentin lysate with focus on TGF-beta signaling. Sci. Rep..

[31] Nasirzade J., Alccayhuaman K.A.A., Kargarpour Z., Kuchler U., Strauss F.J., Panahipour L., Kampleitner C., Heimel P., Schwarz F., Gruber R. (2021). Acid Dentin Lysate Failed to Modulate Bone Formation in Rat Calvaria Defects. Biology.

[32] Kuchler U., Rybaczek T., Dobask T., Heimel P., Tangl S., Klehm J., Menzel M., Gruber R. (2018). Bone-conditioned medium modulates the osteoconductive properties of collagen membranes in a rat calvaria defect model. Clin. Oral. Implant. Res..

[33] Schafer S., Viswanathan S., Widjaja A.A., Lim W.W., Moreno-Moral A., DeLaughter D.M., Ng B., Patone G., Chow K., Khin E. (2017). IL-11 is a crucial determinant of cardiovascular fibrosis. Nature.

[34] Ng B., Dong J., D’Agostino G., Viswanathan S., Widjaja A.A., Lim W.W., Ko N.S.J., Tan J., Chothani S.P., Huang B. (2019). Interleukin-11 is a therapeutic target in idiopathic pulmonary fibrosis. Sci. Transl. Med..

[35] Adami E., Viswanathan S., Widjaja A.A., Ng B., Chothani S., Zhihao N., Tan J., Lio P.M., George B.L., Altunoglu U. (2021). IL11 is elevated in systemic sclerosis and IL11-dependent ERK signalling underlies TGFbeta-mediated activation of dermal fibroblasts. Rheumatology.

[36] Widjaja A.A., Singh B.K., Adami E., Viswanathan S., Dong J., D’Agostino G.A., Ng B., Lim W.W., Tan J., Paleja B.S. (2019). Inhibiting Interleukin 11 Signaling Reduces Hepatocyte Death and Liver Fibrosis, Inflammation, and Steatosis in Mouse Models of Nonalcoholic Steatohepatitis. Gastroenterology.

[37] Widjaja A.A., Viswanathan S., Shekeran S.G., Adami E., Lim W.W., Chothani S., Tan J., Goh J.W.T., Chen H.M., Lim S.Y. (2022). Targeting endogenous kidney regeneration using anti-IL11 therapy in acute and chronic models of kidney disease. Nat. Commun..

[38] Dong B., Hiasa M., Higa Y., Ohnishi Y., Endo I., Kondo T., Takashi Y., Tsoumpra M., Kainuma R., Sawatsubashi S. (2022). Osteoblast/osteocyte-derived interleukin-11 regulates osteogenesis and systemic adipogenesis. Nat. Commun..

[39] Pountos I., Walters G., Panteli M., Einhorn T.A., Giannoudis P.V. (2019). Inflammatory Profile and Osteogenic Potential of Fracture Haematoma in Humans. J. Clin. Med..

[40] Kidd L.J., Stephens A.S., Kuliwaba J.S., Fazzalari N.L., Wu A.C., Forwood M.R. (2010). Temporal pattern of gene expression and histology of stress fracture healing. Bone.

[41] Zhang H., Wang R., Wang G., Zhang B., Wang C., Li D., Ding C., Wei Q., Fan Z., Tang H. (2021). Single-Cell RNA Sequencing Reveals B Cells Are Important Regulators in Fracture Healing. Front. Endocrinol..

[42] Sakai R., Eto Y. (2001). Involvement of activin in the regulation of bone metabolism. Mol. Cell Endocrinol..

[43] Sakai R., Eto Y., Hirafuji M., Shinoda H. (2000). Activin release from bone coupled to bone resorption in organ culture of neonatal mouse calvaria. Bone.

[44] Inman G.J., Nicolas F.J., Callahan J.F., Harling J.D., Gaster L.M., Reith A.D., Laping N.J., Hill C.S. (2002). SB-431542 is a potent and specific inhibitor of transforming growth factor-beta superfamily type I activin receptor-like kinase (ALK) receptors ALK4, ALK5, and ALK7. Mol. Pharmacol..

[45] Nishina T., Deguchi Y., Miura R., Yamazaki S., Shinkai Y., Kojima Y., Okumura K., Kumagai Y., Nakano H. (2017). Critical Contribution of Nuclear Factor Erythroid 2-related Factor 2 (NRF2) to Electrophile-induced Interleukin-11 Production. J. Biol. Chem..

[46] Timmermans R.G.M., Bloks N.G.C., Tuerlings M., van Hoolwerff M., Nelissen R., van der Wal R.J.P., van der Kraan P.M., Blom A.B., van den Bosch M.H.J., Ramos Y.F.M. (2022). A human in vitro 3D neo-cartilage model to explore the response of OA risk genes to hyper-physiological mechanical stress. Osteoarthr. Cartil. Open.

[47] Zhu X., Cheng J., Yu J., Liu R., Ma H., Zhao Y. (2023). Nicotinamide mononucleotides alleviated neurological impairment via anti-neuroinflammation in traumatic brain injury. Int. J. Med. Sci..

[48] Ochando J., Ordikhani F., Boros P., Jordan S. (2019). The innate immune response to allotransplants: Mechanisms and therapeutic potentials. Cell. Mol. Immunol..

[49] Berasain C., Avila M.A. (2014). Amphiregulin. Semin. Cell Dev. Biol..

[50] Vaidya M., Lehner D., Handschuh S., Jay F.F., Erben R.G., Schneider M.R. (2015). Osteoblast-specific overexpression of amphiregulin leads to transient increase in femoral cancellous bone mass in mice. Bone.

[51] Jay F.F., Vaidya M., Porada S.M., Andrukhova O., Schneider M.R., Erben R.G. (2015). Amphiregulin lacks an essential role for the bone anabolic action of parathyroid hormone. Mol. Cell. Endocrinol..

[52] Yang H., Zhu J., Guo H., Tang A., Chen S., Zhang D., Yuan L., Liu G. (2022). Molecular cloning, characterization, and functional analysis of the uncharacterized C11orf96 gene. BMC Vet. Res..

[53] Johnston J., Ramos-Valdes Y., Stanton L.A., Ladhani S., Beier F., Dimattia G.E. (2010). Human stanniocalcin-1 or -2 expressed in mice reduces bone size and severely inhibits cranial intramembranous bone growth. Transgenic Res..

[54] Kamata T., So T.Y., Ahmed Q., Giblett S., Patel B., Luo J., Reddel R., Pritchard C. (2020). Fibroblast-Derived STC-1 Modulates Tumor-Associated Macrophages and Lung Adenocarcinoma Development. Cell Rep..

[55] Shi M., Yuan Y., Liu J., Chen Y., Li L., Liu S., An X., Luo R., Long D., Chen B. (2018). MSCs protect endothelial cells from inflammatory injury partially by secreting STC1. Int. Immunopharmacol..

[56] Sjarif D.R., Ploos van Amstel J.K., Duran M., Beemer F.A., Poll-The B.T. (2000). Isolated and contiguous glycerol kinase gene disorders: A review. J. Inherit. Metab. Dis..

[57] Walker A.P., Muscatelli F., Stafford A.N., Chelly J., Dahl N., Blomquist H.K., Delanghe J., Willems P.J., Steinmann B., Monaco A.P. (1996). Mutations and phenotype in isolated glycerol kinase deficiency. Am. J. Hum. Genet..

[58] Qin A., Cheng T.S., Pavlos N.J., Lin Z., Dai K.R., Zheng M.H. (2012). V-ATPases in osteoclasts: Structure, function and potential inhibitors of bone resorption. Int. J. Biochem. Cell Biol..

[59] Lyons R.M., Keski-Oja J., Moses H.L. (1988). Proteolytic activation of latent transforming growth factor-beta from fibroblast-conditioned medium. J. Cell Biol..

[60] Carnes D.L., Maeder C.L., Graves D.T. (1997). Cells with osteoblastic phenotypes can be explanted from human gingiva and periodontal ligament. J. Periodontol..

[61] Kraus D., Wolfgarten M., Enkling N., Helfgen E.H., Frentzen M., Probstmeier R., Winter J., Stark H. (2017). In-vitro cytocompatibility of dental resin monomers on osteoblast-like cells. J. Dent..

[62] Robey P.G., Termine J.D. (1985). Human bone cells in vitro. Calcif. Tissue Int..

[63] Zhong Y., Zhang X., Chong W. (2022). Interleukin-24 Immunobiology and Its Roles in Inflammatory Diseases. Int. J. Mol. Sci..

[64] Singh B., Carpenter G., Coffey R.J. (2016). EGF receptor ligands: Recent advances. F1000Research.

[65] Hua K.C., Feng J.T., Yang X.G., Wang F., Zhang H., Yang L., Zhang H.R., Xu M.Y., Li J.K., Qiao R.Q. (2020). Assessment of the Defatting Efficacy of Mechanical and Chemical Treatment for Allograft Cancellous Bone and Its Effects on Biomechanics Properties of Bone. Orthop. Surg..

[66] Pruss A., Gobel U.B., Pauli G., Kao M., Seibold M., Monig H.J., Hansen A., von Versen R. (2003). Peracetic acid-ethanol treatment of allogeneic avital bone tissue transplants—A reliable sterilization method. Ann. Transplant..

