# Supplementary figures and images for: Bone Allograft Acid Lysates Change the Genetic Signature of Gingival Fibroblasts
Source: Int J Mol Sci. 2023 Nov 10;24(22):16181. doi: 10.3390/ijms242216181 (PMC10671348; doi:10.3390/ijms242216181)

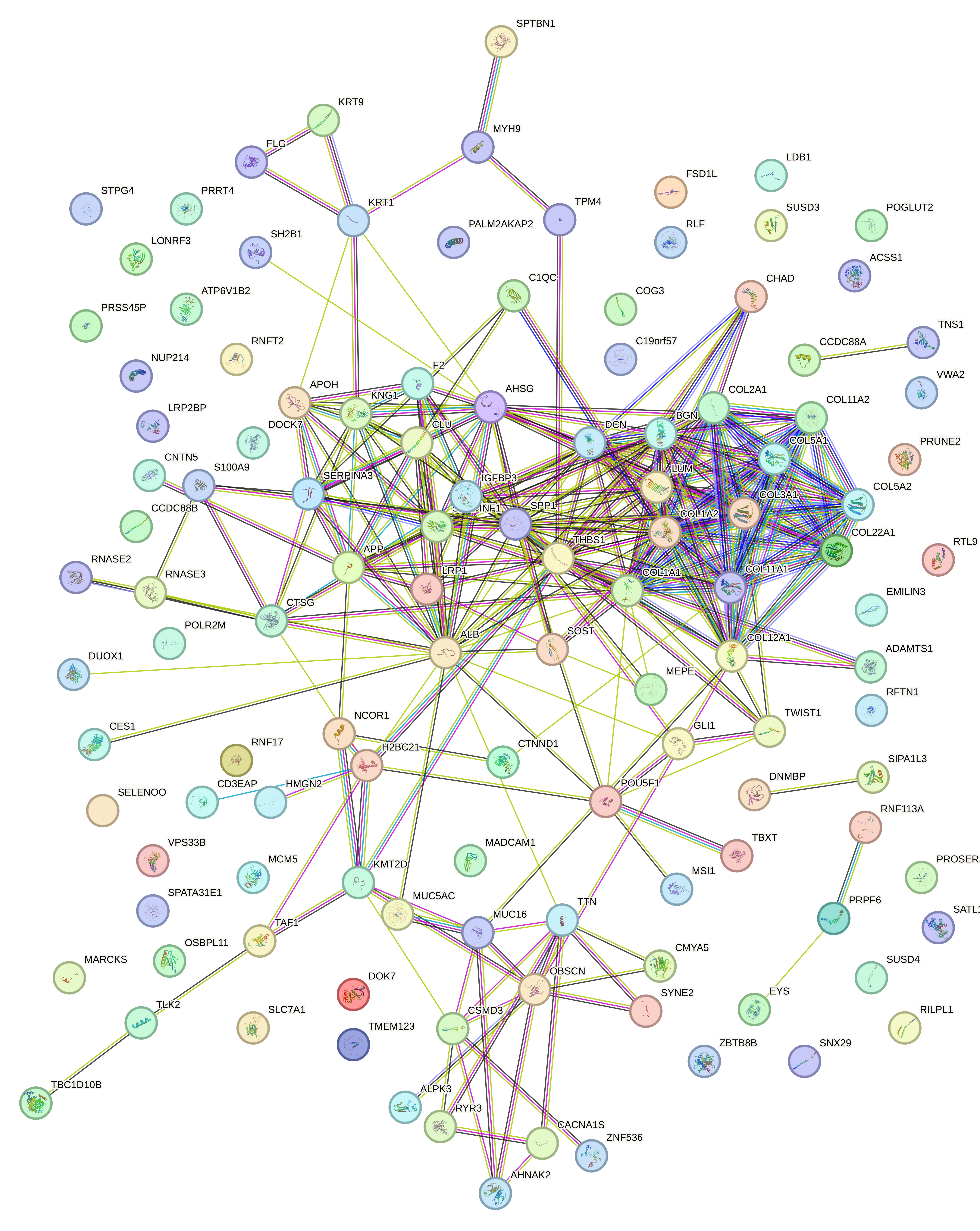

Supplement: Supplementary file 1 [file ijms-24-16181-s001.zip › ijms-2685056-supplementary/Supplementary Files/Proteomics_Supl Tables 2/String analysis of 122 proteins/string_hires_image.png]

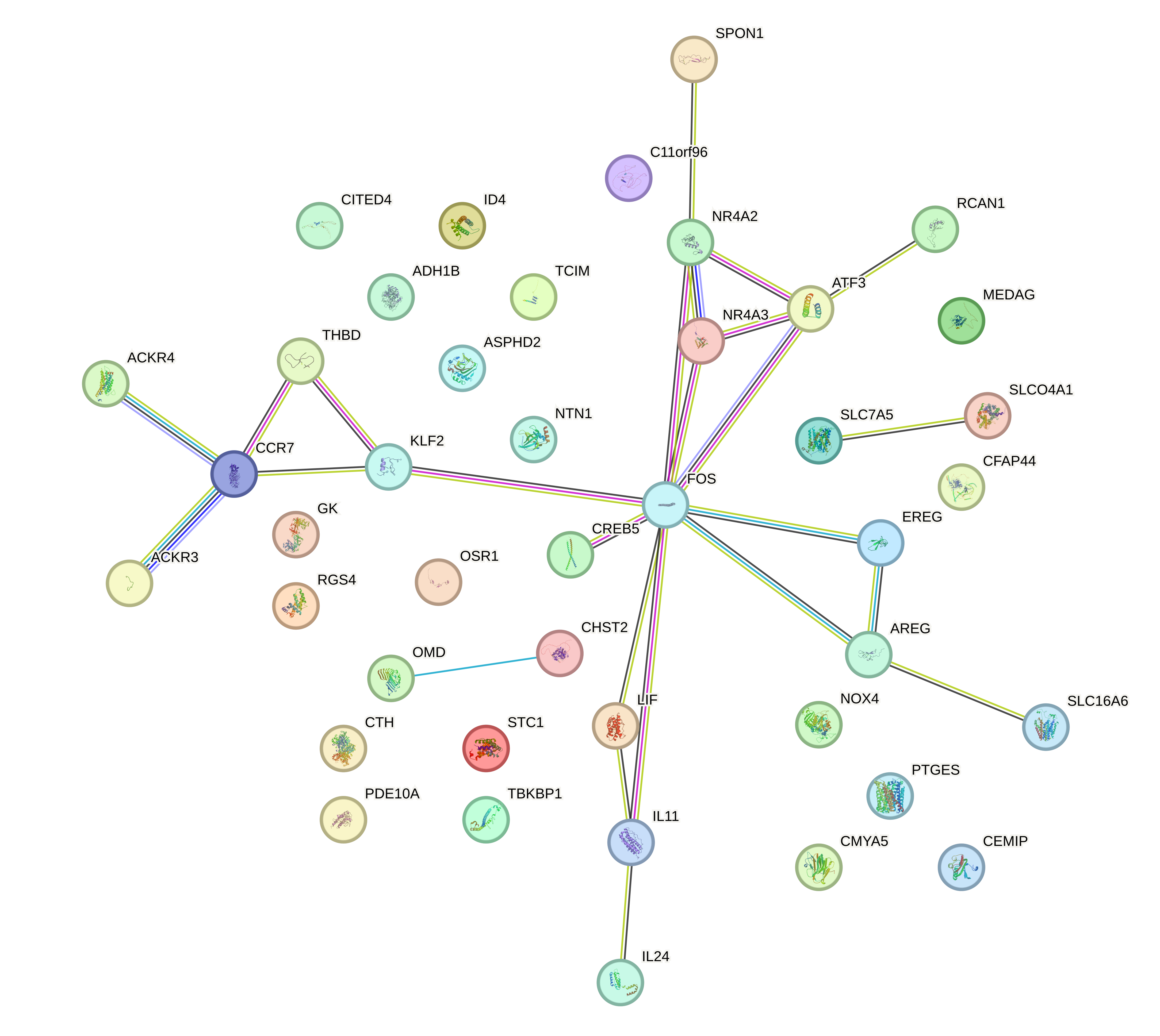

Supplement: Supplementary file 1 [file ijms-24-16181-s001.zip › ijms-2685056-supplementary/Supplementary Files/RNAseq_Suppl Tables 1/String analysis/String of strongly regulated genes.png]
